# Drought and Oxidative Stress in Flax (*Linum usitatissimum* L.) Entails Harnessing Non-Canonical Reference Gene for Precise Quantification of qRT-PCR Gene Expression

**DOI:** 10.3390/antiox12040950

**Published:** 2023-04-18

**Authors:** Prasanta K. Dash, Rhitu Rai, Sharat Kumar Pradhan, Sheelavanta Matha Shivaraj, Rupesh Deshmukh, Rohini Sreevathsa, Nagendra K. Singh

**Affiliations:** 1 ICAR-National Institute for Plant Biotechnology, Pusa Campus, New Delhi 110012, India; 2 ICAR-National Rice Research Institute, Cuttack 753006, India; 3 Indian Council of Agricultural Research, Krishi Bhawan, New Delhi 110012, India; 4 Department of Biotechnology, Central University of Haryana, Mahendragarh 123031, India

**Keywords:** flax/linseed, drought, reference genes, *Actin*, *EF1a*, *UBQ*, ROS, ROIs, cellular oxygen

## Abstract

Flax (*Linum usitatissimum* L.) is a self-pollinating, annual, diploid crop grown for multi-utility purposes for its quality oil, shining bast fiber, and industrial solvent. Being a cool (Rabi) season crop, it is affected by unprecedented climatic changes such as high temperature, drought, and associated oxidative stress that, globally, impede its growth, production, and productivity. To precisely assess the imperative changes that are inflicted by drought and associated oxidative stress, gene expression profiling of predominant drought-responsive genes (AREB, DREB/CBF, and ARR) was carried out by qRT-PCR. Nevertheless, for normalization/quantification of data obtained from qRT-PCR results, a stable reference gene is mandatory. Here, we evaluated a panel of four reference genes (*Actin*, *EF1a*, *ETIF5A*, and *UBQ*) and assessed their suitability as stable reference genes for the normalization of gene expression data obtained during drought-induced oxidative stress in flax. Taking together, from the canonical expression of the proposed reference genes in three different genotypes, we report that *EF1a* as a stand-alone and *EF1a* and *ETIF5A* in tandem are suitable reference genes to be used for the real-time visualization of cellular impact of drought and oxidative stress on flax.

## 1. Introduction

Flax (*Linum usitatissimum* L.) belongs to the family Linaceae and is a diploid (2n = 30), self-pollinating, shrub grown as an annual crop for its oil and fiber. It also finds mention in ancient agricultural civilization constituting the “Neolithic package” of crops that were domesticated for oil/fiber around 10,000 years ago [1,2]. However, through modern agricultural husbandry, flax was developed to an economically important field crop which is grown not only for agricultural produce but also for obtaining industrial solvent. The cultivated flax is a dual source of cellulose-rich bast fiber and high-quality oil. The fine quality fibers obtained from the bast/stem are used in the textile industry for manufacturing luxurious linen clothes, whereas seeds (linseed) are used to extract oil that is rich in omega-3 fatty acids. The extracted oil is recommended for human consumption as a dietary supplement [3]. Consumption of linseed oil has immense health benefits as it contains anti-inflammatory, anti-carcinogenic, and antioxidant compounds along with nutraceuticals [4,5]. The oil is also a rich source of a precursor of multiple phytoestrogens and antioxidants with reported health benefits. It is also used as a solvent in industrial products such as paints, varnishes, linoleum, and ink for printing [6]. Even the left-over cake of linseed after extraction of oil, i.e., linseed meal, is used in livestock feed that serves as a rich source of proteins for cattle [6]. These high agri-commercial utilities make flax a multi-purpose remunerative crop for farmers, rendering farming a successful venture.

Plants are sessile organisms and, to avoid climate-induced biotic and abiotic stresses, have developed intrinsic adapted mechanisms for successful completion of life. However, climatic and edaphological changes directly impact plant cellular activity. Flax being an annual crop, has exceptional edaphological adaptive capabilities toward moderate warm and cool climatic conditions. The crop grows better in moist loam soil because of its shallow root system [7], but is vulnerable to dry spells and high temperature [8]. Amongst other abiotic stresses, drought stress drastically affects flax seedling growth, flowering, seed development, and yield [8]. Spells of intermittent drought stress also directly interfere with crop-stand in field condition while prolonged exposure to drought leads to partial reduction of cellular oxygen resulting in the production of reactive oxygen intermediates (ROIs), also known as active oxygen species (AOS), or reactive oxygen species (ROS) [9]. The over-production of ROIs can lead to rapid oxidation which results in disruption of cell membranes and cell components and eventual cell death [9]. Drought also triggers accumulation of ROS and increases cellular oxidative stress leading to loss of economic harvest i.e., oil, fiber, and nutraceuticals in flax.

Globally, France, Russia, and China are the top producers of flax fiber while India, Canada, and China are the top producers of linseed oil [1,8]. The drought stress in flax that arises either out of irregular rainfall or a decreased ground water table or limited water availability or rise in ambient temperature [10] is the leading factor impeding global flax production and productivity. Therefore, there is an impetus to decipher the molecular, cellular, and physiological impact of drought and oxidative stress on flax [10].

Although drought-responsive gene (DRG) expression changes have been elucidated in model plant systems [10,11,12] and priority crops [13,14], meagre progress has been made to understand the regulatory mechanism in flax during drought and associated oxidative stress. Due to self-pollinating biology, short vegetative stage, enriched genomic information, and suitable transformation protocol [15,16,17], flax is an ideal plant for functional genomic and genetic studies. While the marker assisted breeding has been successfully implemented to develop new varieties of flax, the functional genomics through next-generation sequencing (NGS) informatics and gene expression profiling helped to divulge the regulatory stress genomics. Coupled with the whole genome sequence information [3], the transcriptome data [8,18], and small-scale genomics study [19] have generated minimum essential genomic information that have paved a path for large-scale functional genomic analysis to understand molecular and regulatory mechanisms of drought and oxidative stress in flax. The last few years have witnessed a massive increase in the genomics data of flax achieved through mRNA transcript profiling, microarray analysis [5], and quantitative real-time PCR (qRT-PCR) [20] showing transcriptome dynamics to stresses in real-time [21]. However, to visualize the precise dynamic changes at the cellular level, validation of target gene expression through qRT-PCR is essential, for which selection of stable reference genes (RGs) is a prerequisite [22]. The RGs help to normalize and measure the precise expression levels of mRNA transcripts [22,23]. Thus, it is critical to identify suitable RGs, which are expressed ubiquitously through multiple tissues/organs in flax and independent of experimental variation [23].

Since the expression of RGs must be independent of experimental variation, the choice of RG/RGs differ for a specific experiment/stress condition [24,25]. Ideally, the housekeeping genes or cytoskeleton genes or steadily expressing genes are selected as RGs. However, studies have shown that these genes are expressed inconsistently in different cell types during different developmental stages of plants or under different experimental conditions [26,27]. Therefore, it is mandatory to evaluate a set of selected RGs under desired experimental conditions to obtain a reproducible and accurate qRT-PCR result [28] that is reflective of precise cellular dynamic changes.

There are reports about single and multiple gene expression analysis through Northern blot [29,30,31] and classical RT-PCR [32] in flax. Although different types of RGs are used, some of them are found suitable for wide-range of experiments due to their stable expression pattern, such as GAPDH, cyclophilin and 18S rRNA, actin, *EF1a*, ubiquitin, and beta tubulin [33,34,35,36,37]. However, few studies have been carried for the normalization of gene expression using qRT-PCR in flax [30,38] and by selecting distinct RGs for different experimental conditions in other species [1,39,40]. However, no stable RGs are selected and calibrated for qRT-PCR experiments in flax during drought and drought-induced oxidative stress conditions. Therefore, we evaluated the expression of some of the previously reported RGs and validated them through statistical methods to be used as correct calibrators in flax for dynamic quantification of drought and associated oxidative stress.

Choosing an appropriate reference gene for the normalization of target gene expression data is difficult, especially when studying gene expression using RT-PCR assays under different stress conditions such as drought stress. Normalization of target gene expression against reference genes reduces the variability caused by differences in RNA extraction, reverse transcription, and PCR efficiency between samples and ensures that any observed changes in target gene expression levels are not due to experimental artefacts. The choice of an appropriate reference gene, on the other hand, is critical, because the expression levels of many commonly used reference genes can vary significantly under different experimental conditions. Given the critical role of selecting appropriate reference genes, our study was designed to investigate the expression of four commonly used reference genes including *Actin*, *EF1a*, *ETIF5A,* and *UBQ* and evaluate their utility for use as reference genes in gene expression studies under drought stress using RT-PCR assays. By examining the expression stability of these four genes under drought stress conditions, we aimed to identify the most reliable and consistent reference genes that can accurately normalize gene expression data and reduce experimental variability.

## 2. Materials and Methods

### 2.1. Sequence Identification, Retrieval and Primer Designing

The selected gene sequences (AREB1, AREB2/ABF4, DREB1/CBF, DREB2, ARR1I; Table 1) were retrieved from the TAIR database (https://www.arabidopsis.org/; accessed on 12 August 2022) and used for the identification of their orthologs using CDS sequences of *Linum usitatissimum* (v1.0) from an annotated scaffold assembly at phytozome database (https://phytozome.jgi.doe.gov/pz/portal.html, accessed on 12 August 2022) using BLAST. The top hit of the blast result with maximum identity, highest bit score, and lowest e-value were selected. The specific primers were designed for the selected housekeeping genes (Actin, EF1a, ETIF5A, and UBQ; Table 2) and for drought-responsive gene orthologs in flax. The primers were designed using Primer3 software keeping parameters for amplicon size 100 bp to 150 bp, optimal Tm at 60 °C, with 40–60% GC content.

### 2.2. Plant Material, Total RNA Isolation, and cDNA Synthesis

Flax cultivar Hira, Mukta, and R552 were obtained from All India coordinated research project (Linseed), Kanpur, and grown for several generations in the laboratory and stored for research purposes. The seeds were germinated in vermiculite pots and the plants were grown in a growth chamber under 16 h day (20 °C) and 8 h night (18 °C) conditions for uniform growth. The whole plants were harvested 21 days after sowing and frozen immediately in liquid nitrogen and stored at −80 °C. Drought treatment to the flax cultivars was given as mentioned previously [8]. Total RNA was extracted using the plant RNA isolation kit and DNA contamination was eliminated through on-column DNAse I treatment. RNA purity was assessed by determining the OD_260_/OD_280_ and OD_260_/OD_230_ ratios. Isolated total RNA was used for the cDNA synthesis using first strand cDNA synthesis kit according to the manufacturer’s protocol and subsequently dissolved and stored in nuclease-free water.

### 2.3. qRT-PCR Conditions and Analyses

The qRT-PCRs were carried out in 96-wells plates using SYBR Green in a reaction volume of 20 μL (5 μL diluted cDNAs, 10 μL of 2× SYBR Green mix and primer pairs at 0.4 μM). All PCR reactions were performed under the following conditions: 95 °C for 15 min, 40 cycles of 10 s at 95 °C and 30 s at 60 °C. For each primer pair, a melting curve was generated in order to confirm the specificity of the amplification and the PCR products were checked on a 4% agarose gel. Each experiment was carried out using three biological replicates of each analyzed sample. The specific amplification of the selected genes was confirmed by melting curve analysis having a single peak. For data analysis, the relative expression of drought-responsive genes was analyzed using the 2^-ΔΔCt^ method. *EF1a* was used as RG. ΔΔCt was calculated as the difference between ΔCt of sample (Ct of drought-responsive gene- Ct of RG).

### 2.4. Statistical Analyses of RGs Expression, Stability, and Their Validation through Drought-Responsive Genes

The qRT-PCR expression results provided in the form of Ct values, were used for the calculation of statistical significance through NormFinder software (https://moma.dk/normfinder-software accessed on 12 August 2022). As NormFinder ranks the stability of the tested genes independent to each other by using model-based variance estimation, it was used to rank the best suited RG for normalization in qRT-PCR [41]. To validate the significance of screened RG, the relative expression of each gene amongst the selected drought-responsive genes was calculated using the selected RG in three biological replicates for each sample.

## 3. Results

### 3.1. Selection of Candidate RGs and DRGs and Their Amplification

The usage of different housekeeping genes for the normalization of gene expression data in specific experimental conditions is a routine but essential requirement to obtain precise, reproducible results in molecular biology experiments. After reviewing the literature, we selected a panel of four housekeeping genes *Actin*, *Elongation Factor 1-α* (*EF1a*), *Eukaryotic translation initiation Factor 5A* (*ETIF5A*), and *Ubiquitin* (*UBQ*) from different functional classes which exhibited stable expressions [1,26,27,28]. Similarly, a panel of reported drought-responsive genes such as *Abscisic-acid Response Element Binding Protein1* (*AREB1*), *AREB2*/*ABRE binding factor 4* (*AREB2*/*ABF4*), *Dehydration-Responsive Element-Binding1*/*C-repeat Binding Factors* (*DREB1*/*CBF*), *DREB2A,* and *Arabidopsis response regulator1* (*ARR1*) were taken for commensurate validation of the selected RGs [42,43,44,45]. The protein sequences of the above selected genes from *Arabidopsis* were retrieved and used to search homologs in a flax (genome) database using BLAST with the aforementioned parameters. We set a stringent E-value cut-off (2.006–49) to obtain the best target genes wherein the percent identity ranged from 42 to 60%. The five top-hit flax sequences (Table 1) obtained from the BLAST result were further selected and gene-specific primer pairs were designed (Table 2) for qRT-PCR analysis.

**Figure 1:**
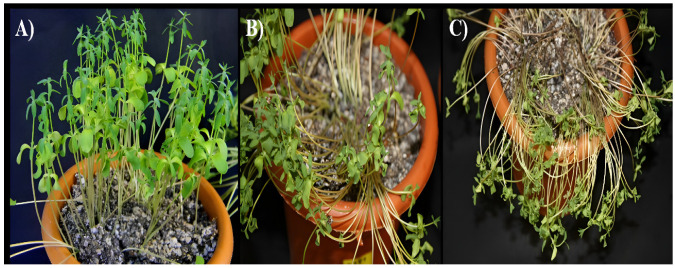
Drought stress induction and appearance of drought and oxidative stress symptoms in flax. (**A**) Unstressed control plants, (**B**) four-day stressed plants, and (**C**) five-day stressed plants showing visible symptoms of drought. The plants were grown at 16 h/20 °C (day) and 8 h/18 °C (night) for 21 days before induction of stress.

**Figure 2:**
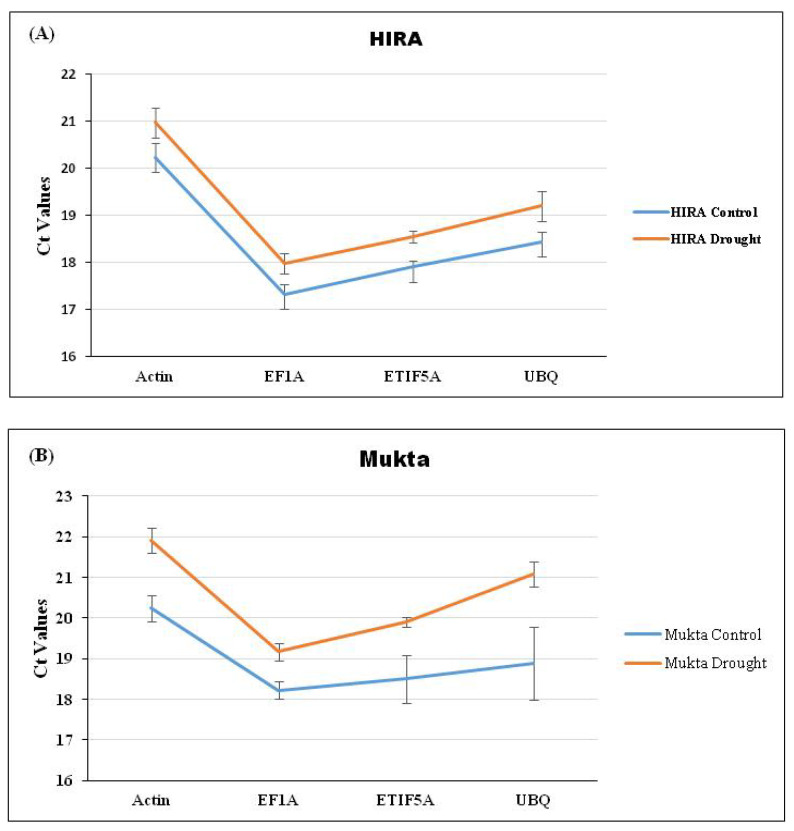

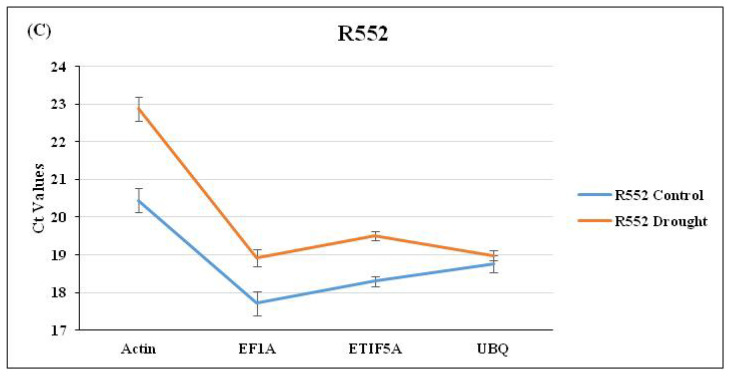
Expression analysis of four selected RGs (*Actin*, *EF1a*, *ETIF5A*, and *UBQ*) through qRT-PCR analysis in (**A**) Hira, (**B**) Mukta, and (**C**) R551 cultivars of flax.

Drought was induced as per a standard protocol [4,8] until the visible symptoms were observed in plants (Figure 1). The expression of selected RGs was checked through qRT-PCR using three flax cultivars viz. Hira, Mukta, and R552 by imparting drought stress (Figure 1). The average Ct values for each RG were calculated for control as well as drought stress separately for the three cultivars. We observed the lowest (and constant) Ct value of 17.19 for the control and 17.63 for drought stress (Figure 2A), respectively, for *EF1a* in Hira. Similarly, *Actin* also exhibited a constant Ct value of 20.28 (control) and 20.88 (drought) in comparison to *ETIF5A* and *UBQ* RGs (Figure 2A) in cultivar Hira. We also observed a minimal standard deviation (±0.047) for *Actin*, although *EF1a* had lowest Ct value (Figure 2A) of 17.19. Likewise, *EF1a* exhibited the lowest and constant Ct value of 18.24 (control) and 19.2 (drought) with a standard deviation of ±1.06 (Figure 2B) in the Mukta cultivar. *UBQ* exhibited a high deviation in Ct values of 18.8 (control) and 21.1 (drought) in the Mukta cultivar (Figure 2B) with a high standard deviation (±2.06). In cultivar R552, we observed the lowest Ct value for *EF1a,* i.e., 17.4 and 18.7 in the control and drought, respectively. Nonetheless, the Ct values of *UBQ* also exhibited less deviation of 18.7 in the control and 19.0 in droughtstress (Figure 2C) with minimal standard deviation (±0.04).

### 3.2. Analysis of Stability of Expression of Selected RGs

As it is mandatory to normalize the expression of RGs in qRT-PCR experiments, this was accomplished by NormFinder that normalizes expression data statistically and ranks the stability of RGs to be considered for precise expression profiling. Thus, the Ct values of the RGs from all the three cultivars from drought and unstressed conditions were discerned into four subgroups and stability scores were computed from NormFinder. The subgroup HM represented Hira-Mukta, MR represented Mukta-R522, HR represented Hira-R522, and HMR represented single group of Hira-Mukta-R522 cultivars. Most of the subgroups showed *ETIF5A* (0.008–0.018) and *EF1a* (0.005–0.008) as the two best stand-alone genes with the best stability scores (Table 3). These results of stability score indicated that *ETIF5A* and *EF1a* are also of stable expression.

Further, the Ct values of combinations of each RGs were taken as a single group (HRM). When all the subgroups taken together were considered as single group (HRM), *EF1a* exhibited the best stability score of 0.007 followed by *ETIF5A* with a score of 0.015 (Table 3). As per the NormFinder results, we concluded that the RGs *ETIF5A* and *EF1a* are the best two genes with stability scores of 0.07 and 0.015, respectively, whereas individually the best stability score for *ETIF5A* was 0.008 followed by 0.005 obtained for *EF1a* (Table 3). Nonetheless, the *UBQ* (0.025) was found to be the least stable RG when all the Ct values were taken as a single group (HMR). It was also observed to be the least stable RG with a stability score of 0.016, 0.033, and 0.023 when the subgroups were computed individually (Table 3).

Further, we calculated the stability score for a pair of selected RGs as normalization using a single reference gene is rarely justified [46]. Since UBQ was observed to exhibit the lowest stability score in the individual subgroups and single group, it was excluded from evaluating the best possible RGs pairs. It was observed that a pair of RGs in each individual group showed stability that ranged from 0.009 to 0.019 (HM), 0.012 to 0.021 (MR), and 0.006 and 0.026 (HR) (Table 4) while the combination of EF1a and ETI5A exhibited the best stability score of 0.010 when all the subgroups were taken as one group (HMR).

### 3.3. Validation of RGs through Expression of DRGs

The expression analysis using statistical methods to identify a suitable RG to be used for the normalization of the qRT-PCR results must be commensurate with the expression of canonical drought-responsive genes in flax. Thus, it is required to check the authenticity/suitability of a selected RG for its coordinated expression and/or whether it helps in the normalization of the expression of DRGs during drought and oxidative stress. Therefore, we used the selected RG *EF1a* for commensurate expression profiling of the five canonical drought-responsive genes (DRGs) in flax such as *AREB1*, *AREB2*/*ABF4*, *DREB1*/*CBF*, *DREB2A,* and *ARR1*.

**Figure 3:**
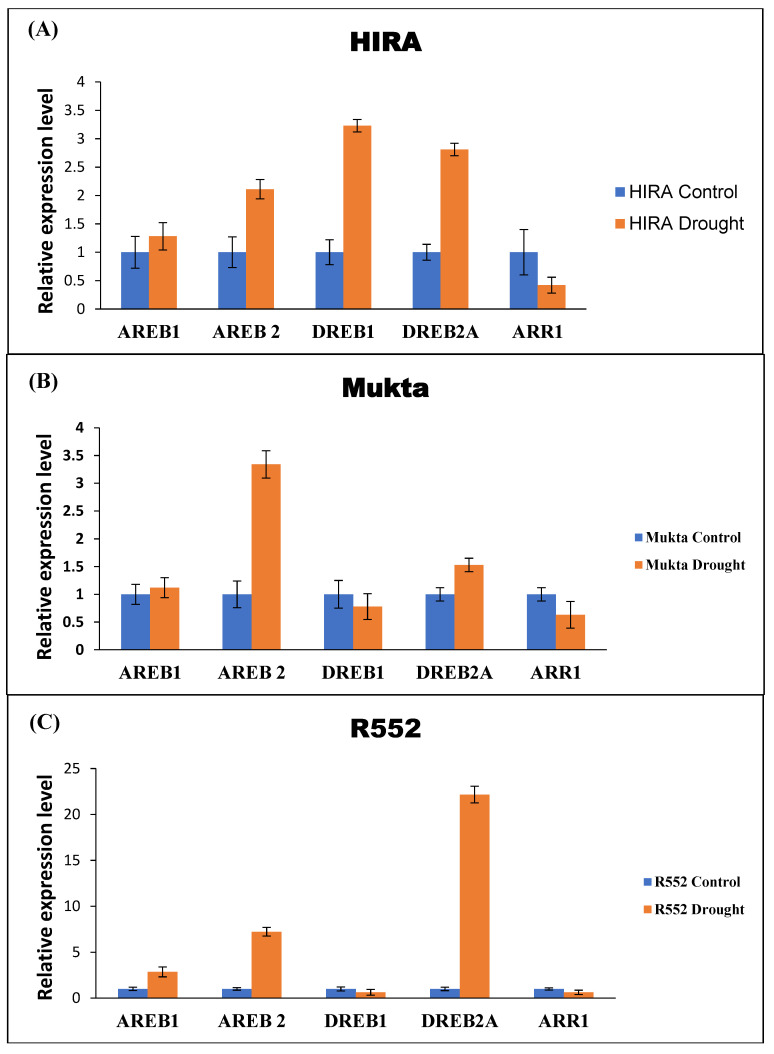
Expression analysis of selected five DRGs with respect to *EF1a* in three cultivars of flax. Error bars indicate ±SE of three biological replicates.

The identified DRGs in flax were used for obtaining the qRT-PCR expression data after imparting drought stress. The expression profiling of all five DRGs were computed by relative quantification using *EF1a* as a reference gene. The results suggest that *AREB2* has a higher expression while *ARR1* has the lowest uniform expression (Figure 3A) in all three cultivars. It was further observed that *DREB1* and *DREB2A* have higher expression in Hira, whereas they are antagonistically expressed in Mukta and R552 cultivars (Figure 3). In Mukta, *DREB1* exhibited 0.2-fold lower expression while *DREB2A* recorded a 0.3-fold higher expression (Figure 3B). Surprisingly, *DREB1* exhibited 0.2-fold lower expression while *DREB2* exhibited 20-fold higher expression in the R552 genotype (Figure 3C).

## 4. Discussion

Over the last decade, the advent of new genomic tools has hastened research with the generation of enormous biological data [46,47,48,49,50,51] with high accuracy and efficiency [52,53,54,55,56]. These techniques have also enabled visualization of the impact of experiments/reactions in real-time. Among these technologies, fluorescent dye-based techniques have become some of the most powerful methods in molecular biology which are used for identification, localization, and expression of biomolecules in living systems. Second to fluorescent dye-based techniques, qRT-PCR has become the other pivotal technology amongst biologists for its high sensitivity, specificity, dynamic range, and the capability to detect extremely low-expressed genes in a given condition with high precision [57]. Further, it expedites concomitant gene expression profiling (multiplexing) with multiple experimental conditions using various tissue samples/treatments [20]. Although qRT-PCR provides multiple advantages for gene expression analysis, intrinsic variations (mRNA concentrations and efficiency of reverse transcription) affect the end result of qRT-PCR [22]. Thus, an error correction measure is always adopted to compare the expression level by normalizing to a standard. Canonically, adequate normalization is accomplished by using internal reference genes (RGs), habitually called the housekeeping genes, as they are unaffected by imposition of stress or any intrinsic experimental variation. The normalization of observed experimental results is carried out by relative quantification of stable RGs with the target genes. Although all species possess RGs actively involved in the metabolic activity, their expression varies in different species. Earlier reports suggest that same housekeeping genes are not expressed uniformly in two different species [1] and the traditional reference genes exhibit substantial variation under given circumstances [46]. Therefore, it is obligatory to check systematic validation and the expression of RGs in different plant systems for the normalization of gene expression data in a given experimental set-up. It is also a prudent experimental practice to select stable RGs that are independent of genotypic variation by selecting multiple cultivars, taking into account intra- and intergroup variation of RGs to evaluate expression stability.

Through this investigation, we selected a panel of four RGs (*Actin*, *EF1a*, *ETIF5A,* and *UBQ*) belonging to different classes of drought-responsive genes to be used as RG for the normalization of gene expression data. Our selection of RGs, however, is specific to gene expression data obtained in drought and oxidative stress experiments in flax. Further, to ascertain the best stability amongst the selected RGs, all the obtained Ct values of individual experiments were taken as a single group (to generate a robust dataset) and further divided into subgroups to compute through NormFinder (Table 3). Most of the combinations of the results revealed *ETIF5A* and *EF1a* as stable expressed RGs (Table 4). As of today, multiple studies using *EF1a* as RG for normalization of the gene expression data in many species [58,59,60] have been carried out. Nevertheless, few studies have reported the use of Eukaryotic Translation Initiation Factor (*ETIF*) genes as a suitable RG [61]. However, it has also been reported that this class of gene is disqualified as a single internal control gene and is not suitable to be considered as an RG [62,63] for its inconsistency. Therefore, based on our results, we selected *EF1a* as an RG for normalization of the expression analysis of drought-responsive genes (DRGs) in flax.

Photophilic plants respire oxygen (O_2_), a molecule essential for all organisms involved in aerobic cell metabolism, as it is the only process leading to the formation of ATP [64]. It has been estimated that 3% of the cellular oxygen [65], however, is converted into reactive oxygen species (ROS) that hinder cellular homeostasis affecting physiological and biochemical conditions such as tolerance to drought, salt, and oxidative stress that deters plant growth, development, and productivity. Comprehensive studies have also been carried out to decipher the molecular mechanisms of drought-responsive gene regulation responding to various molecular [66,67], biochemical, and physiological changes [9,68] in several crop plants including flax [69]. However, to validate the stable expression of selected RG *EF1a* during drought stress in flax, expression analysis was conducted with selected four DRGs as their expression is specifically induced during drought and associated oxidative stress. Further, to check the suitability of *EF1a* for precise quantification and reproducible biological interpretation, expression analysis was conducted using three flax cultivars Hira, Mukta, and R552. The inclusion of three cultivars differing in multiple agronomic traits gave the advantage of selecting a robust, stable, and precisely expressing RG that is independent of genotypic variation. Our results revealed *EF1a* is the most suitable stand-alone gene while a combination of *EF1a* and *ETF5A* in tandem are the best RG pairs to be used for normalization of qRT-PCR results for drought stress experiments in flax.

This study on the genome-wide identification of drought-induced gene expression has been carried out in flax [8] to authenticate commensurate expression of selected RG with reported drought-responsive genes. For this, we selected *AREB1*, *AREB2*/*ABF4*, *DREB1*/*CBF DREB2A,* and *ARR1* genes. To ensure the selection of DRGs are generic/wide-range *AREB1*, *AREB2*/*ABF4*, *DREB1*/*CBF*, and *DREB2A* were selected that are TFs while *ARR1* belonging to the broad class of response regulators [45] was selected. The TFs *AREB1* and *AREB2*/*ABF4* belong to the class of ABA-responsive genes and along with *AREB3* are cooperatively up-regulated to bind to the ABA-responsive element during abscisic acid signaling for adaptation to oxidative and water stress [69]. Similarly, *DREB1*/*CBF* and *DREB2A* belong to the TFs *APETALA2*/*Ethylene Response Element Binding Factors* (*AP2*/*ERF*) family that are involved in a myriad of plant developmental processes during abiotic stresses [70]. Earlier studies suggested that *DREB1*/*CBF* is induced by the cold, whereas *DREB2A* is induced by dehydration and high-salt stresses [71,72]. Further, *ARR1* belongs to *Arabidopsis* type B response regulators (ARRs) which, independent of cytokinin induction, regulate many drought- and ABA-responsive genes [45]. Previous studies have shown that *ARR1*, *ARR10*, and *ARR12* act redundantly as negative regulators in the process [45].

As per the obtained qRT-PCR results, we conclude that *AREB2* is highly expressed in all three cultivars during the imposition of drought stress. Similarly, *DREB2A* is also highly expressed in Mukta and R552 while *DREB1* is highly expressed in Hira. Consequently, we found that *ARR1* had the lowest expression (Figure 2) in all the three cultivars similar to the earlier reports [45]. Taken together, the cumulative findings from all these results suggests that *EF1a* is the cardinal gene with stable, consistent expression and is suitable to be used as a reference gene (RG) for the normalization of gene expression data during drought stress experiments in flax because the expression profiling of DRGs (taking *EF1a* as reference gene) is commensurate with their canonical expression. Thus, *EF1a,* the most suitable RG in our drought experiment might be used for the normalization of the qRT-PCR expression results during drought and oxidative stress experiments in flax. However, in the absence of a single, robust, unique universal reference gene, the selection of RGs must be documented for each experimental setup and tailored to each activation process.

## 5. Conclusions

The identification of reference genes for the normalization of gene expression data in plants exposed to specific conditions is an essential requirement to understand gene regulation. Although differential expression of drought-responsive genes (DRG) has been elucidated in model plant systems such as Arabidopsis and priority crops such as rice, meagre progress has been made to understand the regulatory mechanisms of DRGs in flax under drought stress. To address these gaps in the present study, we identified better candidate reference genes which can be used for gene expression analysis in flax under stress. Upon evaluation of a panel of reference genes for their suitability for normalization of gene expression, we found that *EF1a* as a stand-alone while *EF1a* and *ETIF5A* in tandem qualify for real-time estimation of gene expression changes in flax exposed to drought and oxidative stress. Further, the expression of drought-responsive genes was analyzed using the *EF1a* as an RG. The study indicated that among the DRGs, *AREB2* is highly expressed in all three cultivars viz. Hira, Mukta, and R552 under drought stress. Through this investigation, we present candidate RGs that can be used for gene expression analysis in flax under stress. The RGs and the information about DRGs provided here will further aid in understanding the molecular mechanisms underlying drought tolerance among different flax cultivars.

## Figures and Tables

**Table 1:** Drought-responsive gene orthologs *AREB1*, *AREB2/ABF4*, *DREB1/CBF*, *DREB2A*, and *ARR1* in flax with their gene ID, E-value, and % identity taking *Arabidopsis* as reference.

Arabidopsis		*Linum usitatissimum*				
Gene Name	Gene Id	Gene Id	E-Value	Bit Score	%Identity	CDS Length (in bp)
*AREB1*	AT1G45249	Lus10006489	5.00 × 10^−86^	315	50.35	1209
*AREB2/ABF4*	AT3G19290	Lus10014066	2.00 × 10^−104^	376	50.76	1266
*DREB1/CBF*	AT4G25490	Lus10031657	9.00 × 10^−59^	223	60.59	696
*DREB2A*	AT5G05410	Lus10034902	2.00 × 10^−49^	193	42.29	753
*ARR1*	AT3G16857	Lus10037719	6.00 × 10^−177^	618	52.16	2121

**Table 2:** List of primer pairs used for validation of reference genes (RGs) and drought-responsive genes (DRGs) in qRT-PCR expression analysis in flax.

Sr. No.	Name	Sequence (5′-3′)
1	LuActin_qRTFwd	TCCAGGCCGTTCTTTCTCTA
2	LuActin_qRTRev	CTGTAAGGTCACGACCAGCA
3	LuEF1A_qRTFwd	GCTGCCAACTTCACATCTCA
4	LuEF1A_qRTRev	GATCGCCTGTCAATCTTGGT
5	LuETIF5A_qRTFwd	TGCCACATGTGAACCGTACT
6	LuETIF5A_qRTRev	CTTTACCCTCAGCAAATCCG
7	LuUBQ_qRTFwd	CTCCGTGGAGGTATGCAGAT
8	LuUBQ_qRTRev	TTCCTTGTCCTGGATCTTCG
9	LuAREB1_qRTFwd	ATCAGATGGGATTGGGAAGAGC
10	LuAREB1_qRTRev	GGAGGCAGAAGAGAATGCTCA
11	LuAREB2_qRTFwd	TGTTGAGAGAAGACACAGAAGG
12	LuAREB2_qRTRev	GGAGATGAATGAAGAACTGGAG
13	LuDREB1_qRTFwd	CGGCGGTGGAAGCGACGAC
14	LuDREB1_qRTRev	GCCGGGGCTTTTGACGAGCA
15	LuDREB2A_qRTFwd	AGACGTTAAGGACTATGAGTGGC
16	LuDREB2A_qRTRev	GGCTTGCTGTTAGGGGATAATA
17	LuARR1_qRTFwd	CAAGGCAATATTGAGGTGGGCTC
18	LuARR1_qRTRev	CTCTGCTGCTGGCGTGGAACA

**Table 3:** Stability of the selected RGs in multiple flax cultivars. Stability scores of RGs were computed from the Ct values obtained from qRT-PCR data of drought stressed flax plants by using NormFinder. The groups are the combination of flax cultivar Hira-Mukta (HM), Mukta- R552 (MR), and Hira-R552 (HR), and Hira-Mukta-R552 (HMR).

Group	HMR	HM	MR	HR
Gene Name	Stability Score			
*Actin*	0.020	0.012	0.030	0.019
*ETIF5A*	0.015	0.016	0.018	0.008
*EF1A*	0.007	0.007	0.008	0.005
*UBQ*	0.025	0.016	0.033	0.023

**Table 4:** Computation of stability scores for combinations of two RGs for flax qRT-PCR gene expression study. The best pair of RGs was obtained by calculating an individual stability score using NormFinder. The groups are the combination of flax cultivars Hira (H), Mukta (M), and R552 (R).

Group	Best Combination of Two Genes	Stability Score
HMR	*EF1a* and *ETIF5A*	0.010
	*EF1a* and *Actin*	0.016
	*ETIF5A* and *Actin*	0.018
HM	*EF1a* and *ETIF5A*	0.009
	*EF1a* and *Actin*	0.011
	*ETIF5A* and *Actin*	0.019
MR	*EF1a* and *ETIF5A*	0.012
	*EF1a* and *Actin*	0.021
	*ETIF5A* and *Actin*	0.021
HR	*EF1a* and *ETIF5A*	0.006
	*EF1a* and *Actin*	0.014
	*ETIF5A* and *Actin*	0.026

## Data Availability

Not applicable.
